# Integrating care for neurodevelopmental disorders by unpacking control: A grounded theory study

**DOI:** 10.3402/qhw.v11.31987

**Published:** 2016-09-07

**Authors:** Gustaf Waxegård, Hans Thulesius

**Affiliations:** 1 Department of Psychology, Linnaeus University, Växjö, Sweden; 2 Research Unit, Region of Kronoberg, Department of Clinical Sciences, Family Medicine, Lund University, Lund, Sweden

**Keywords:** Autism, ADHD, health services, integrated care, neurodevelopmental disorders, care pathways

## Abstract

**Background:**

To establish integrated healthcare pathways for patients with neurodevelopmental disorders (ND) such as autism spectrum disorder and attention-deficit hyperactivity disorder is challenging. This study sets out to investigate the main concerns for healthcare professionals when integrating ND care pathways and how they resolve these concerns.

**Methods:**

Using classic grounded theory (Glaser), we analysed efforts to improve and integrate an ND care pathway for children and youth in a Swedish region over a period of 6 years. Data from 42 individual interviews with a range of ND professionals, nine group interviews with healthcare teams, participant observation, a 2-day dialogue conference, focus group meetings, regional media coverage, and reports from other Swedish regional ND projects were analysed.

**Results:**

The main concern for participants was to deal with overwhelming ND complexity by *unpacking control*, which is control over strategies to define patients’ status and needs. *Unpacking control* is key to the professionals’ strivings to expand constructive life space for patients, to squeeze health care to reach available care goals, to promote professional ideologies, and to uphold workplace integrity. Control-seeking behaviour in relation to ND unpacking is ubiquitous and complicates integration of ND care pathways.

**Conclusions:**

The *Unpacking control theory* expands central aspects of professions theory and may help to improve ND care development.

Forming successful care pathways to improve care quality and accessibility for patients with neurodevelopmental disorders (ND) is a major challenge for health care and society. Integrated care pathways have been defined as “…management technologies which formalise multidisciplinary team-working and enable professionals to examine their roles and responsibilities” (Allen, Gillen, & Rixson, 2009) and as “…structured multidisciplinary care plans which detail essential steps in the care of patients with a specific clinical problem” (Campbell, Hotchkiss, Bradshaw, & Porteous, 1998).

Dominant ND-diagnoses, as defined in major professional classification systems like the Diagnostic and Statistical Manual of Mental disorders (DSM-V) (American Psychiatric Association, 2013), are autism spectrum disorder (ASD) and attention deficit hyperactivity disorder (ADHD) with an estimated prevalence of about 1% and 5%, respectively (Baird et al., 2006; Lai, Lombardo, & Baron-Cohen, 2014; Matthews, Nigg, & Fair, 2013; NICE, 2008; Polanczyk, De Lima, Horta, Biederman, & Rohde, 2007; Zablotsky, Pringle, Colpe, Kogan, Rice & Blumberg 2015). These are complex psychological and medical conditions, associated with a high degree of psychiatric comorbidity (Gillberg et al., 2004; Goldstein & Schwebach, 2004; LoVullo & Matson, 2009; Matson & Nebel-Schwalm, 2007; Rao & Landa, 2014).

The presentation of ND in individuals is characterized by profound heterogeneity, and the level of functional impairment caused by ND varies from mild to severe (Hobson, 2014; Nigg, 2013; Ronald & Hoekstra, 2011; Sharp, McQuillin & Gurling, 2009; Shevell 2010; Wåhlstedt, Thorell, & Bohlin, 2009). ND can have a negative impact on family relations and are associated with reduced quality of life, especially if left untreated (Karst & Van Hecke, 2012; Kooij et al., 2010). The notion of heterotypic continuity, meaning that the same underlying difficulty can express itself differently at different points in an individual's development, is important in ND (Rutter, 2013a; Rutter & Sroufe, 2000). Though not completely understood and the target of much sociological critique (e.g., Pajo & Cohen, 2013; Timimi et al., 2004), ND diagnoses are considered as reasonably valid entities by experts in the field (Antshel et al., 2007; Antshel, Phillips, Gordon, Barkley, & Faraone, 2006; NICE, 2008; Remschmidt et al., 2005; Rutter, 2005a, 2005b, 2005c).

While having a certain degree of utility for certain purposes, psychiatric diagnoses have been criticized more generally to lack validity (Kendell & Jablensky, 2003). They are crude descriptive categories and provide only a fraction of the insights needed to provide adequate care for patients with ND. As a consequence, mental health care has moved towards transdiagnostic, dimensional frameworks in several areas (e.g., Clark, 2009; Ellard, Fairholme, Boisseau, Farchione, & Barlow, 2010; Mansell, Harvey, Watkins, & Shafran, 2009). In the ND field also, there is awareness of internal inconsistencies in the diagnostic categories as historically used (e.g., Caye et al., 2016; Willcutt et al., 2012; Witwer & Lecavalier, 2008), and today's diagnostic systems may turn out to be partially invalid as knowledge on the causes of ND advances. The use of “ND” rather than specific differential diagnoses in this paper reflects the authors’ cautious stance on the usefulness of categorical differential diagnostics as the foundation of ND care. It also reflects a view of ND as a set of often interrelated and overlapping conditions, mirroring a range of difficulties that constitute meaningful targets for a designated care structure.

However conceptualized, detection, assessment, and treatment of ND require the skills and services of several healthcare professions and healthcare tiers, putting care pathways to the test.

Known barriers to receiving proper ND care are related to a wide variety of factors, from the characteristics of individual patients to society-wide factors and politics (Elsabbagh et al., 2014; Wright et al., 2015). Concerns have been voiced about under-diagnosing as well as over-diagnosing of ND (Moynihan, Doust, & Henry, 2012; Sayal, Taylor, Beecham, & Byrne, 2002), late detection of particularly ASD (Bryson, Rogers, & Fombonne, 2003; Chakrabarti, Haubus, Dugmore, Orgill, & Devine, 2005; Kleinman et al., 2008), and a general poor accessibility to care and information for children as well as adults with ND (De Vries, Glavina, Major, Mayern, & Shellshear, 2007; Mansell & Morris, 2004; Matheson et al., 2013; Miller et al., 2008).

The needs of children with ND are typically multivariate (i.e., they may need pharmacological, educational, psychotherapeutic as well as social/economical support). Alas, health and community services for people with ND are often experienced as poorly integrated (Griffith, Totsika, Nash, & Hastings, 2012; Miller et al., 2009; Osborne & Reed, 2008).

Cautious optimism has been expressed that clinical care pathways could increase the quality in health care (Deneckere et al., 2012; Panella, Marchisio, & Di Stanislao, 2003), and there is expert consensus on the need for well-established care pathways for children and adolescents with ND (Carbone, 2013; Kendall, Taylor, Perez, & Taylor, 2008). Integrating health care has generally proved challenging and opposed forces such as increased specialization and decentralization of health care, and conflicting motives for integrating care among stakeholders acts against it (Åhgren, 2012; Trägårdh & Lindberg, 2004). Transitions between units in health care for young people with ND are fraught with problems, the transition from child and adolescent to adult mental health services being the most studied (Belling et al., 2014; Hall et al., 2013; Swift et al., 2013).

Provider characteristics play a central role in creating accessible and coherent ND care pathways (Chiri & Warfield, 2012; Fiks, Hughes, Gafen, Guevara, & Barg, 2011; Foy & Earls, 2005; Sayal, Goodman, & Ford, 2006). Professionals hold considerable power to define the lives and care of persons with intellectual disability such as ND (Punzi, Erlandsson, & Lundin, 2016; Tideman & Svensson, 2015) and have been called Lords of the dance (Scott, 2008)—prime choreographers of modern institutions, which motivates in-depth studies exploring the integrating process from the professional perspective. The purpose of this classic grounded theory (CGT) study was to conceptualize the major problems encountered by professionals when organizing ND care pathways and to find out how these problems are resolved, since few such studies have been undertaken. The research question was: “What is going on in care pathways for neurodevelopmental disorders?”

## Methods

### Data collection

Efforts to manage and develop a care pathway for ND in a Swedish region with a population of 190,000 were studied for a period of approximately 6 years. The first author was analytic manager for a nationally funded project with the goal to improve ND care pathways and was auditing a regional care process for ND in children and adolescents. This main case was complemented with data from external care pathways for purposes of theoretical sampling. The data sources for the study are described below.

Forty-two individual interviews with professionals were carried out, with 34 women and 8 men from 24- to 71-years-old; among them 15 were psychologists, 8 physicians, 5 nurses, 3 social workers, 3 occupational therapists, 2 physiotherapists, 2 special educators, 1 was a healthcare developer, 1 economist, and 2 were patient representatives. Ten of the interviews were theoretically sampled from two other Swedish regions. Participants were initially asked to share their experiences from working in an ND care pathway, with the aim to elicit incidents of relevance for the research question cited above. Interviews were structured only by the research question but gradually constrained by the emerging theory and its categories according to CGT. The interviews were performed by the first author at the professionals’ workplaces and typically lasted an hour. Nine group interviews, following the same principles, were performed with healthcare *teams* concerned with ND, representing different care levels and specialties. Glaser explicitly states that CGT interviews shall not be recorded and transcribed. We adhered to this principle as a rule, but recorded a subset of interviews for the sake of correct quotes and to make them available to both authors. These recordings were not transcribed in detail but used for field notes in the same way as the other interviews. In addition, a clinical focus group consisting of three psychologists (including Waxegård, 1st author), one educational therapist and one occupational therapist met bi-weekly at 40 occasions. Focus-group meetings always revolved around the research question and discussions were coded by the authors. Members were drawn from different workplaces, facilitating organizational outreach and understanding of professional concerns not immediately recognized by the authors. Apart from working as clinicians and interacting with the care pathway on a daily basis, members documented aspects of the care pathway and functioned as a think-tank on ND issues.

An external national expert on care pathway development was consulted to arrange a 2-day dialogue conference on ND care pathways for health professionals, managers, and healthcare developers. The 65 conference participants—a large part of the professionals working in the care pathway—jointly analysed care pathway issues in small multi-professional, cross-clinic groups. Barriers and facilitators to care pathway development were discussed from the perspectives of professional co-action, co-ordination, and shared or non-shared understanding of ND. Small-group reflections were documented on flipcharts, coded, and memoed.

Statistical data were collected on trends in the number and proportion of ND-related visits in the care pathway, proportion of ND-related visits between the various clinics assessing and treating ND, type of and number of ND-related diagnoses across the regions municipalities, and sex differences in assigned diagnoses in the care pathway. Data were extracted in co-operation with the regional unit for analytic support. National statistics from the Swedish Board of Health and Welfare was used to document national trends and variability in diagnoses and medication. ND-related local routines, clinical guidelines, policies, and frequent collaboration partners were documented and turned into field notes and memos according to the CGT method, see below. Media coverage, defined primarily as the dominant regional newspaper and public service television, of ND care was studied. A total of 94 articles spanning the entire study period were coded. Six official reports of on-going projects on ND care pathway development in other Swedish regions were studied and coded. Lastly, actions, such as managerial or team decisions, made by different care process stakeholders were treated as incidents and included as data.

In sum, the full range of professions typically working with ND in Swedish health care provided data to this project. A project report to the Swedish Board of Health and Welfare was written by the first author and was treated as a large theoretical memo in relation to this article.

### Data analysis

In the absence of clearly articulated empirical frameworks to guide research on ND care pathway development, CGT (Glaser, 1978, 1998) was chosen as the method of investigation. It was presumed that CGT openness to several types of data would be necessary to explain a rich phenomenon like an ND care pathway, characterized by a variety of historical, cultural and social artefacts, and constructions. CGT is open to any kind of data to inform an emerging theory, in accordance with the CGT “all is data”-dictum. CGT is generated by an iterative process where data are subject to constant comparison, coded into categories, elaborated in memo writing, and by an imperative to think conceptually, not descriptively. A good CGT is possible to modify in the face of new data, has grab, fit, and relevance for the field under study. The core conceptual pattern discovered in the data should be abstract of time, place, and people, that is, the same conceptual pattern should be possible to identify at another point in time, in another setting and with different participants involved.

In this study, based on data coding of the large number of incidents initially yielding 1401 substantive codes, 47 preliminary categories were conceptualized. Incidents in CGT are retrieved from field notes. A field note is the researcher's documentation of events in the research area, such as interviews or an organizational change. Typically, field notes in this project were written as Word documents which were then coded line by line. Through the processes of constant comparison and sorting of memos, categories were gradually abstracted and eventually reduced to two interrelated core categories with four subcategories, respectively. No software package for qualitative data analysis was used; data were analysed using a word processor and manually with pen and paper.

This paper deals with the core category of *unpacking control* and the subcategories of *expanding constructive space, squeezing, ideologizing*, and *isolating*. The parallel core resolution pattern is called *trust testing*, and is presented in detail elsewhere (Waxegård & Thulesius, 2016). In brief, trust testing is behaviour exploring whether professional unpacking collaboration can occur without being “stuck with the buck” and if other professionals can be approached to solve our own unpacking priorities. The main function of *trust testing* is for professionals to decide on promoting local or collective control over unpacking in the care pathway. *Trust testing* thus regulates the opportunities for integrating the care pathway through collective action and adds to the literature on social dilemmas.

**Figure 1:**
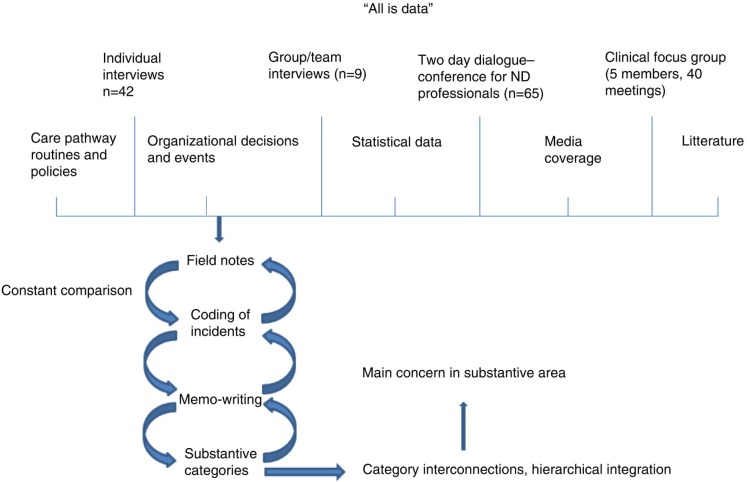
Overview of data and of the classic grounded theory method as applied in this study.

For an overview of data and the CGT method applied in this study, see Figure 1.

### Theory development and theoretical sampling

Theoretical sampling is how CGT deals with theory development and saturation. It propels the CGT forward by asking “what next?” in terms of data gathering, “for what?” in relation to substantive codes and “why?” in relation to analysis of memos (Glaser, 1998, p. 173). The main “what next” in this study quickly became to sample data that helped the theory progress from initial formulations representing the categories of “sprawl” and “fragmentation” in the care pathway. This resulted in an appreciation of the multi-level complexity associated with ND, involving levels of care, leadership issues, professional competition, multi-faceted needs and high level of co-morbidity in patients with ND, societal views on ND, and more. It was realized that ND had to be *(re-)constructed* in the eyes of the care pathway professionals, in relation to a wide and conflicting variety of professional concerns. Such reconstruction seemed to be a social process influenced by more factors than ideal models of “best clinical practice.” Data sampling thus increasingly mirrored the attempts by professionals to impose order on this challenging ND complexity.

The “for what?” then became to try out codes sensitive to this concern. Categories emerged that reflected preferred ways of dealing with ND complexity as well as their motivational sources. A *conceptual elaboration* (Glaser, 1978, p. 40) strongly supported by further data sampling was made at this point: when faced with more complexity than you are able to handle with available resources, you can either adapt the system by raising the ability to handle complexity, or draw a line beyond which no further complexity is taken into account. *Complexity regulation* in relation to ND was found to be the central theoretical code, answering many of the “why?” questions posed to memos. Differences in professionals’ tolerance for complexity cast light on diverse issues such as conflicts over the extent and timing of neuropsychological testing, implementation of or resistance to new clinical paradigms, the sense of arbitrariness as to what constitutes ND diagnoses and the never-ending discussions about patient belonging and referral policies.

Eventually the core category, or main resolution pattern, was conceptualized as “unpacking control,” since taking control over ways to define patients and their needs (controlling patient flows, assessment routines, professional competence, team structure, and so on) was the prime strategy to handle ND complexity.

### Contribution to theory from data sources

In CGT, the core category is supposed to represent the best fit for most of the data. Since categories were continuously reduced and theoretically integrated, most data sources contributed to most categories albeit with some differences in emphasis. *Interviews* with professionals contributed richly to all categories and are the main foundation of the theory. One category was more informed by interviews than by other data sources, namely the concern of *expanding constructive space* for patients. *Media coverage* contributed most obviously to the subcategories *squeezing*, ideology, trust issues, and dialogue need, and implicitly to the latent pattern of stakeholder competition about methods to unpack ND. *Focus group meetings* made clearer the large number of variables that must be reflected on when constructing care pathways for ND, such as levels of care, specialism vs. generalism, age of the patients, preventive vs. diagnostic frameworks, formal descriptions of clinic responsibilities, healthcare law, profession-specific concerns, budget issues, clinic history and tradition, political decisions, geography, leadership philosophy, and more. *Statistical data* illustrated increased demand for ND care, confirmed the gender difference usually found in ND with boys more often diagnosed than girls, and also clearly illustrated medically unmotivated geographical differences in practices and/or report on practice. In addition to contributing with some hard facts, qualitative analysis of statistic data revealed a poor standard or lack of relevant quantitative measures, increasing general uncertainty about what was actually at hand in terms of ND care. Initial poor record keeping in the care pathway and increased organizational focus on some types of statistic data became evident when external demands for *squeezing* and accountability for queues and availability increased due to national politics in the early phases of the study. Thus, qualitative analysis of statistical data contributed much to the category of *squeezing*. *The dialogue conference* specifically highlighted difficulties in combining a high level of flexibility with high level of structure in the care pathway. Overall, the conference validated the finding of general ND complexity as the main challenge for an ND care pathway as well as unpacking control as the prime means to solve it. *Literature search* provided an academic context to situate the theory and validated the notion of ND complexity.

CGT does not exist in isolation from the wider field of qualitative research (Berterö, 2012, 2015). While qualitative data analysis concepts such as triangulation, reflexivity, audit trail, and peer debriefing do not apply to CGT, they can be applied to enhance the understanding of the methodology and are briefly discussed below.

### Triangulation or all is data

Triangulation (i.e., Smith, 1996, pp. 193–194) corresponds in part to the “all is data”-rule in CGT. Allowing different types of data, qualitative as well as quantitative, to inform the theory helps the researcher to correct faulty inductions due to narrow data sampling. An example of the rich triangulating possibilities afforded by the study design was constant comparison of data on referral practices stemming from policy statements, participant observation, and from formal interviews, respectively.

### Reflexivity

A challenge for the first author was to abandon preconceived professional interpretations of what was going on in the data. To correct a tendency to side with the opinions of experienced clinicians, a reduced affective tone in relation to the analysis was sought as the study progressed. Reflection was necessary to understand the demanding work situation for many of the participants, causing stress, irritability and frustration and a felt pressure on the authors to share these sentiments, to sympathize, and to “buy in” on participants’ stories. A helping thought during reflective efforts was “who in this care pathway does NOT want the best for all patients?” It was then recognized that bad intentions or disinterest among participants was not hindering care pathway development. This insight renewed the interest in what the participants were *actually doing*. Seeing that, in spite of different viewpoints among participants, they were still engaging in similar processes gave confidence that the authors had reached an understanding that was separable from preconceived impulses.

### The audit trail

Keeping an audit trail, as described in Lincoln and Guba (1985) is one way of establishing the trustworthiness of any study based on naturalistic inquiry, although this is disputed by Glaser (2004). The audit trail of the present study includes field notes, memos, hypothesized categories and pieces of the emergent theory in the form of Word documents, images, hand-written memos, powerpoint images, e-mails, flipchart documents, excel files and of printed or digital literature, booklets, and policies.

**Figure 2:**
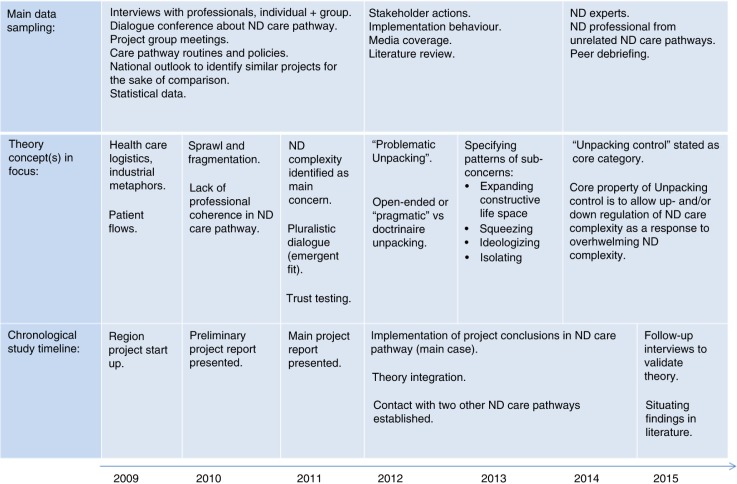
Condensed presentation of data sampling, theory development, and chronological study timeline.

Figure 2 outlines study progress in three areas: data sampling, theoretical concepts in focus, and chronological timing of study events. Milestones in the audit trail were the two project reports written, reflecting the emerging theory. Both reports were distributed broadly in the ND care pathway. Conclusions and inductions made from the dialogue conference were submitted to all participants. Results were presented at several managerial levels and theory contents in some respects informed organizational policy. We believe that the study had transparency with possibilities for participants to provide feedback and follow theory development.

### Peer debriefing

The theory was presented at several formal scientific seminars and informal academic meetings, ensuring input from research colleagues. The theory was also scrutinized in a formal seminar by experienced psychologists specializing in different healthcare areas and naïve to the theory. Comments and criticisms were treated as new data and included in the process of constant comparison.

### Ethics

Kronoberg County Ethics Review Board approved the project in 2009. To explore the challenges professionals in the care pathway collectively face, by way of sharing reflections on professional practice with researchers who were also clinicians was deemed not to pose any major threats to participants’ integrity. No experimental manipulation occurred and the interviews did not ask for any other effort on behalf of the participants than the voluntary sharing of experiences concerning organization and provision of ND care. CGT aims at integration of all data into one theory and at not exposing any specific individual's contributions to the theory, though anonymous quotes may serve as illustrations of the theory. Sampling data from several different care pathways and from multiple data sources over an extended period of time was considered to be of value to reassure participants that any facet of the theory presented is grounded in many observations and not relying on any one interview or statement.

Exposing detailed demographic or other personal information about participants is discouraged in CGT since the theory, not the individual participants’ views, is what matters. This was also one of the reasons why CGT was chosen as a framework from the outset, allowing a theoretical contribution without unduly exposing individual professionals.

## Results

### Unpacking control to solve the main problem of ND complexity

The grounded theory emerging from this study states that *ND complexity overload* is the main problem for professionals in ND care, and that the main strategy to resolve this problem is to establish *unpacking control* in the ND care pathway. *Complexity* in this theory refers to a large number of intricately interacting variables that are demanding to sort out and to represent formally. It reflects the ND properties of not being simple but multi-faceted, hard to disentangle and therefore to fully grasp.

*ND unpacking control* is defined as control over care pathways such as strategies, structures, and methods to understand and define ND symptoms in patients. To *unpack* is, in the transferred sense of the word, to pay close attention to and examine something in detail. *Unpacking control* allows professionals choose to the type and level of complexity that is allowed to emerge when formalizing descriptions of ND. The need to regulate ND care complexity is manifested in primarily four professional sub-concerns: *expanding constructive life space* for individual patients, *squeezing* ND care to increase patient turnover, *ideologizing*, meaning to promote certain professional ideologies or paradigms, and *isolating*, that is to uphold a degree of isolation of the own workplace or team from the rest of the care pathway to ensure structural and social workplace integrity.

To illustrate, *unpacking control* may be converted into more complex assessment procedures to match the complexity of the presenting patient, increasing precision in understanding the patient's problems. Such understanding can inform patient-specific interventions that expands constructive life space.

On the other hand, *unpacking control* can be used to cut back on assessment routines to increase patient turnover and achieve available healthcare goals (squeezing), thus down-regulating care complexity.

Furthermore, *unpacking control* gives the opportunity to strengthen a certain professional ideology or paradigm, ranging from managerial New Public Management ideals to ND-specific or psychotherapeutic models. Such ideologized *unpacking control* can tune up or down ND complexity depending on the contents of the paradigm.

**Figure 3:**
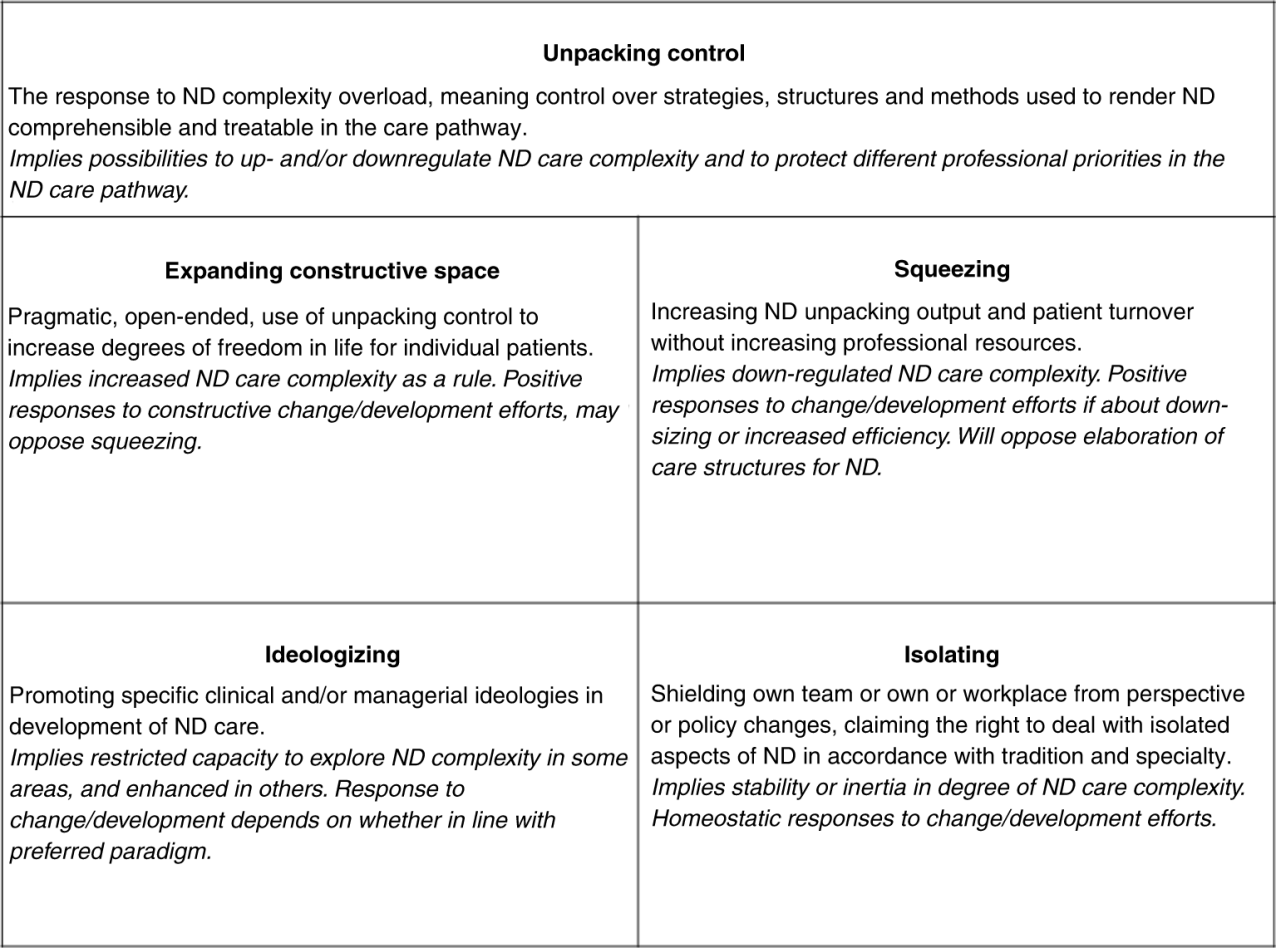
Overview of the unpacking control theory.

Finally, *unpacking control* is instrumental in isolating the own workplace or team to protect it from intrusion or collapse when faced with stressors such as new patient categories or policy changes external to the team or workplace. Such *isolating* of practices implies *status quo* in unpacking complexity, and inertia or homeostatic responses to suggested changes in care pathway policies. For an overview of the theory's main concepts, see Figure 3.

*Unpacking control* is thus a versatile and strategically important tool to impose order on ND complexity. In a process of explicit ND care pathway integration efforts, *unpacking control* becomes particularly relevant to ND professionals and managers, seeking to protect several conflicting core values at once. Without *unpacking control*—the power to influence healthcare strategies, structures, and methods—professionals lose effective ability to tackle ND issues.

In what follows, three main sources of ND complexity are outlined. Then, *unpacking control* strategies in relation to the four professional sub-concerns mentioned above in ND care pathway integration are conceptualized. Two high-impact examples of competition between high- and low-complexity unpacking are provided, before a general discussion.

### ND complexity as main concern

ND-related complexity stems from ND itself, the healthcare organization, and society.

As to ND itself, professionals alluded to the heterogeneity of ND between and within individuals, such as:

It can be sort of heavy, when most assessments are complex, when there is no real clarity at all, and when all observations are pointing in different directions, and there is just sprawl. That can be confusing … as well as difficult to convey to parents.

Or:

Working with ND is very different [from previous job as a ward nurse]… You have to think more for yourself and find your own solutions. It is difficult, really difficult! You have to be much more well prepared for each child that you see, what earlier information there is and so.

Explicit awareness of the objective complexity of ND, encompassing genetic heterogeneity, large phenotypic variability, gene×environment interactions and heterotypic continuity varied between participants but most expressed in some way that ND-intrinsic complexity affected their everyday work and efforts to organize the care pathway.

The healthcare organization contributes with further complexity. Constructing the ND care pathway as one seamless pool of resources for children and families was impossible due to what we term *organizational under-conceptualizing*. This refers to a failure to include one or more vital organizational aspects into action plans, thereby eroding chances of successful care pathway integration. In the example below, a professional illustrates a social process of diagnostic collusion that is difficult to incorporate into a formal policy:

When resources that can benefit patients exist in one diagnose-based clinic and not in the other, it may result in a tendency to “find” the right diagnosis to make the patient fit into the clinic with the more resources.

Participants both pointed to such failures by others to take vital organizational parameters into account, and fell prey to organizational under-conceptualizing in their turn by not distinguishing between care levels, local traditions, personal expertise, and political decisions.

A third source of ND complexity is societal in origin. In the words of one specialist:

… but then the rest of society is not very well-informed … I would say that the largest difficulty is the unevenness of [professional] competence that this patient group faces. There is an incredible amount of prejudice that makes me faint.

Prominent society-wide issues were schools failing to give adequate support, generally negative attitudes towards children with ND and debates over the “true” nature of particularly ADHD. Keeping abreast with regional, national, and international ND care guidelines and scientific advances on causes of and treatment for ND provided additional variables to sort out.

### Unpacking control to expand constructive life space

*Expanding constructive life space* (ECS) is a new concept that emerged in this study and means to unpack the specific limitations and strengths in individual ND patients, taking their life context into account to design interventions that open up new constructive life space. ECS is as much about transforming contextual contingencies in every-day life based on conscientious unpacking as about medical cure and has interesting similarities to the nursing habilitation framework described by Olli, Vehkakoski, and Salanterä (2014). An example would be the special educator who in awareness of a motor-perceptual deficit in a young boy changed the colour and size of a football, thereby helping the boy to enjoy soccer. This improved the relation between him and his distressed father, which was the goal of the intervention.

Unpacking strategies central to the ECS concern are *emphasis on professional craftsmanship*; to encourage a *rich unpacking ecology* in the care pathway, in the form of a broad set of ND-specific unpacking tools (i.e., neuropsychological tests), unpacking strategies (i.e., implementation of national or international ND care guidelines), and unpacking structures (i.e., multi-professional specialized ND teams); and *dialoguing* to elicit unpacking collaboration across clinic and disciplinary boundaries. The use of formal and informal dialogue to raise unpacking complexity constitutes an emergent fit with “pluralistic dialogue,” a grounded theory concept developed by McCallin (2006). *Pluralistic dialogue* is characterized by mentalizing capacity, collaborative leadership, and non-hierarchical interdisciplinary approaches (McCallin, 2003a, 2003b, 2005).

Attending to the needs of individual ND patients seems to propel unpacking complexity upwards, and the ECS focus typically gives a preference for the up-regulation of unpacking complexity in the care pathway. One clinician captured the quality of ECS wonderfully in stating: “Oftentimes, what is needed in ND cases is a bit of special-fix!”

### Unpacking control to squeeze ND care

*Squeezing* is about using *unpacking control* to respond to growing population demands for ND care and to politically set goals about available health care. Here, the concern is to increase unpacking output without upgrading or even by downsizing unpacking resources.

In *squeezing*, unpacking control may be converted into implementation of industrially inspired methods for improving flows and efficiency, such as Lean production, time measurement efforts, Deming's wheel, and other healthcare improvement models. Reflecting a certain lack of strategical consistency, *squeezing* is also characterized by clinics raising one-sided access barriers to ND care. Access barriers provoke similar responses by care neighbours, resulting in ever narrower definitions of the own unit's care responsibilities towards ND patients.

Inside clinics, squeezed unpacking leads to *rationing* of care, where aspects of care are delayed or withheld by *time-stretching* professionals who try to keep up and increase patient turn over. Unchecked *rationing*, such as continuous lack of a specific professional competence in team-based assessment, can lead to erosion of care quality and a collapse of care structures such as specialist teams, reducing complexity in care from the specialist to the generalist level.

*Squeezing* is justified by pointing to reality: demand exceeds resources. Still, this concern easily backfires, since the *squeezing* quest for the lowest possible acceptable level of care signals to professionals that they need to unpack *more* ND symptoms in patients to provide them with care access. *Squeezing* ambitions can cause distrust between professional stakeholders due to the temptation to transfer workload to other clinics under the pretense of collaboration. Since altruistic mutuality is not guaranteed, interclinic collaboration in a *squeezing* context is difficult.

*Squeezing* is the use of *unpacking control* to down-regulate unpacking complexity in ND care—though it yields a constant pending response to re-up-regulate care complexity to reverse the perceived aversive effects of *squeezing*.

### Ideologizing unpacking control

*Unpacking control* may also be used to handle ND complexity by fitting it into a preconceived clinical or managerial paradigm, allowing the professional and the organization to focus on a preselected type and number of ND-related variables when seeing patients. This we call ideological unpacking control.

Differences between professionals in their outlook on some aspects of ND are surprisingly difficult to reconcile and stir emotions when integrating care. Ideological unpacking control flows from policy decisions, is dependent on power structures, and is imposed to restrain some types of professional behaviour to make room for other, more ideologically aligned behaviour. Strategies associated with ideological unpacking are alliance seeking, representation in policy groups, use of status and charisma, falling back on authorities, and identification with professional categories such as professions, teams, or leaders. Non-compliance with policy may also indicate ideologized unpacking.

Rather than any single ideological debate dominating, a number of ideological tensions were discernible. Examples of colliding ideological interests in unpacking control are found between a psychotherapeutic understanding of ND symptoms and seeing ND as functional impairments. It is also seen between the developmental psychological perspective, emphasizing psychosocial prevention, and the medical/psychiatric perspective, emphasizing diagnosis and to some extent medication. To this can be added competitive profession-specific paradigms, as well as competition between what we term professional special-interest clusters which consist of professionals from varying professions but devoted to the same paradigm. Moreover, striking a balance between clinician and managerial unpacking control is emotionally charged and a hotbed for destructive conflicts which may cause a brain drain with many professionals as well as managers quitting their jobs, as seen in our data. Furthermore, a generally ND-friendly discourse can be contrasted to a generally more ND-critical stance, on a dimension ranging from expansive views of ND care to ND being portrayed as even a kind of cuckoo chick in child mental health care. The simplified societal debate about ND and in particular ADHD that oscillates between mother-blame and brain-blame (Partridge, Lucke, & Hall, 2014; Singh, 2004) surfaced in the care pathways as well.

Of more interest to the present study than the content of ideological tensions, however, is the observation that ideological unpacking control is important to professionals and selects what type of ND-related complexity that is allowed to come to the fore.

### Isolating unpacking control

It is not self-evident for a professional or a team to take analytical responsibility for ND beyond traditional expectations at the own workplace. *Isolated unpacking* is to limit professional unpacking responsibility to specific aspects of ND, along with promotion of strictly local unpacking control. This helps to down-regulate ND complexity since changes in perspectives and routines that are warranted when seeing the care pathway as a whole are deflected.

While verbally expressing positive sentiments to remodelling care and “coming together for the best of all children” (*proper lining* in GT terms), actual changes and re-interpretation of organizational boundaries and unpacking habits are often met with isolating actions of resistance and homeostatic strategies to protect the own team or workplace.

Professionals may envision ND care as an “open landscape,” where care resources flexibly travel to patients rather than the opposite. Reality was closer to the metaphor of closed rooms, with expertise being locked in fixed segments of the care pathway.

Shielding the own workplace or team from unwelcome perspective and practice changes when integrating ND care seemed important for several reasons. First, the social solidarity found within but not between teams and clinics is the basis for every day professional work and is not traded lightly for externally defined “improved function” in the ND care pathway. Second, actual threats to workplace integrity arise when developing care pathways. For example, an unfortunate effect of successfully solving workplace tasks is that a team may end up with the unfinished business of other, less productive, units by way of managerial decision. Third, the trend towards increased specialization of care complicates communication between stakeholders. Becoming very good at parts of ND does not automatically transfer to becoming very good at assuming collective responsibility for ND care.

Examples of *isolating* strategies are to simply ignore new collaborative policy, to cut down on inter-clinic dialogue, to engage in a discourse on “core clinical duties,” pointing to tradition, as well as being hypervigilant to collaboration failures.

What is pushed aside in the isolated unpacking strategy is thus the discourse about how to handle multiple perspectives on ND in the care pathway as a whole.

### ESSENCE: too complex to implement

A major illustration of the ambivalence between raised and lowered unpacking complexity in the care pathway is the reception of the ESSENCE idea of child psychiatry advocated by Gillberg (2010). ESSENCE emphasizes on-going neurodevelopment and pragmatic access to early multi-professional pro-active treatment based on documented needs. As such, it challenges the traditional organization for ND care in several ways. In spite of a strong intuitive appeal to many participants and a clear directive to implement it in the care pathway, initial enthusiasm over the ESSENCE concept was gradually worn down when implementation created unanticipated increases in the complexity in care processes. ESSENCE was eventually even banned from organizational discourse by managerial decisions and replaced by “children 0–5.” This replacement of a dense scientific developmental psychopathology concept for a label merely referring to children of a certain age is a prime example of down-regulated unpacking complexity to manage care pathway integration.

### Neuropsychological testing as a burden or rescue

Another poignant example of tensions between high- and low-complexity strategies concerned neuropsychological testing. To exclude extensive neuropsychological testing from unpacking routines could be framed as a harmful simplification of ND complexity, whereas to routinely include it could be seen as burdening the organization without much extra benefit for most patients. Controlling the degree, timing, and impact on interventions of neuropsychological testing seemed to evoke the full range of unpacking discourses, from *ECS* to *squeezing*, *ideologizing*, and *isolating*.

To sum up, to establish ND *unpacking control* is the main strategy for professionals to deal with ND complexity. Real-world unpacking can deviate markedly from the ideal models of ND management in clinical guidelines. What looks on the surface as a mutual interest among professionals in developing the ND care pathway may be better conceptualized as a drive to control unpacking procedures for bolstering often incompatible sub-concerns such as ECS, *squeezing*, *ideologizing*, and *isolating*. Professionals and management are typically either unaware of, or act unaware of, these important but emotionally charged distinctions.

## Discussion

In this study, based on CGT analysis of data from several Swedish regions, professional stakeholders in a care pathway for ND tried to solve the problem of ND complexity by controlling ND unpacking. To unpack ND is to define the status and needs of ND patients using specific strategies, structure, and methods. It includes diagnostic work-up and results in treatment suggestions. *Unpacking control*, with its properties such as *ECS, squeezing, ideologizing*, and *isolating* can be used to raise complexity in care processes or to reduce complexity to manage different professional concerns. Unresolved ND unpacking conflicts can impede the improvements of ND care pathways.

### Explaining unpacking control

The core resolution strategy of unpacking control can be explained by turning to the professions literature. Solving complex tasks with autonomy is a key property of professionalism. As stated by Andrew Abbott, the influential professions scholar: “The central organizing reality of professional life is control of tasks” (Abbott, 1988, p. 84). Unpacking control as conceptualized in this study is similar to Abbott's term *jurisdictional control*, which is to successfully claim ownership of a professional area containing “human problems amenable to expert service” (Abbott, 1988, p. 35). Our finding, unaware of Abbott's definition, that unpacking control is the core professional strategy in the ND care pathway, contributes to the understanding of how professionals uphold jurisdictional control in a changing healthcare landscape.

### Regulation of task complexity matches tenets of professions theory

ND complexity as our study's main concern and the resulting struggle to up-regulate or down-regulate care complexity via *unpacking control* is also a harmonious fit with professions theory. To Abbott, regulation of complexity of tasks is a fundamental aspect of any professional work.

The concept of *professional inference* has particular relevance for the unpacking control theory. Professional inference is used when the connection between diagnosis and treatment is not obvious, which is common in ND. It is a “middle game” of filling in knowledge gaps to solve tasks. An engineer will not just provide the blueprint for the last bridge she repaired when fixing a broken bridge. She will engage in expert calculations, not accessible to lay-men, which are necessary to fix the human problem of not being able to travel this specific bridge.

Some well-established inference methods in the ND care pathway are to employ team-based assessment, or using a range of psychometric instruments to improve on diagnostic predictions about patients. Professional inference is necessary to solve complex tasks, but its’ informal and inaccessible character leaves professionals vulnerable to jurisdictional attacks from other professional groups and from lay-men.

Importantly, to uphold professional jurisdiction, inference methods must neither be too routinized, thereby eliminating the argument for professional problem solving, or too esoteric, thereby failing to provide a clear enough rationale for external stakeholders to accept the procedures. Routine prescription of stimulant medication after a shallow clinical examination is one example of routinization that leads to questioning of psychiatrists’ professional jurisdiction in the ND area. Of more relevance to participants in this study, oversimplification of ND assessment due to *squeezing* pressure is another example of routinizing that threatens the jurisdiction of several professions.

At the other end of the continuum, the lack of implementation success for ESSENCE may be explained by it being an overly esoteric professional inference model. As a broad developmental psychopathology model, ESSENCE covers much but specifies little, giving limited guidance as to how exactly organize care. This undermines professional arguments based on the framework.

### Unpacking control and ontological models

Furthermore, *unpacking control* is more than controlling logistics and flows: it is a way to (re)create the patient in the care pathway, an analytic-constructive effort. Unpacking is creating something new but not inventing arbitrary fictions. This quality of unpacking is aptly captured by the concept of professional ontological models. To Brante (2014, p. 279), a professional structure is “a lasting, self-amplifying relation between a certain type of science, an object and a practice.” The (health) professional act is to redefine a human problem, reducing it to a specific ontological model and thereby rendering it treatable. Focus in ND care pathways is on ND unpacking rather than on ND treatment, we suggest, since unpacking is the key to treatment and to control unpacking is to control treatment: it constitutes the linking of “knowing why” with “knowing how” (Brante, 2014). Unpacking ability, when understood as the application of an ontological model, is necessary to function as a professional and to *ECS* for patients. Therefore, violation of the freedom to unpack leads to professional trust issues.

To elaborate, the ND field can be regarded as linked to an *epistemic culture* as defined by Knorr Cetina (1999). She challenges the idea of a unified science, showing different disciplines even within the “hard” natural sciences to be “epistemic monopolies producing vastly different products” (Knorr Cetina, 1999, p. 4). Within mental health care, several epistemic cultures co-exist. This leads to friction, and *ideologizing* of unpacking control is one conceivable way to overcome the inter-disciplinary tensions surrounding ND concepts. “Unifying” a care pathway may then be equal either to monopolizing understanding of ND in the care pathway, or actively embracing the contribution of different epistemic cultures, making them work together for the best of the patient. Clearly, each strategy has obvious and differing implications for organization of ND care pathways.

### Knowledge gaps fuel the race for professional unpacking control

The ND field is a relatively young discipline. It does not clearly belong to any one profession and the knowledge base is far from complete, leaving ample room for unpacking competition. There are currently controversies as to (a) the true prevalence of ND (Rowland et al., 2015; Stolzer, 2007, 2009; Thomas, Sanders, Doust, Beller, & Glasziou, 2015); (b) the most appropriate instrumentation to diagnose ND (Valo & Tannock, 2010); (c) the stability and validity of ND subtypes and developmental patterns (Lahey, Pelham, Loney, Lee, & Willcutt, 2005; Nigg, Tannock, & Rohde, 2010; Willcutt et al., 2012); (d) how to conceptualize comorbidities (Gargaro, Rinehart, Bradshaw, Tonge, & Sheppard, 2011; Van Steensel, Bögels, & De Bruin, 2013); (e) how to weigh the relative influences of nature and nurture in ND; and (f) how to understand the *interactions* of nature and nurture (Halasz & Vance, 2002; Rutter, 2013b).

In a still broader perspective, the psychological birth of the child is a recent event. Ellen Key proclaimed the 20th century as “the century of the child” (Key, 1900), but up until the 19th century children with behavioural problems were seen as inherently evil or possessed by Satan (Mash & Dozois, 2003, p. 7). Children's brains were in psychiatry's early days thought to be protected from insanity because of their supposed immaturity, and the idea that children can suffer from psychiatric problems still remains controversial (Angold & Egger, 2004, pp. 125–130). Models of child mental health problems have largely been extrapolated from adult models. In a repetition of the pattern, young children's psychopathologies are generally less researched than those of older children (Egger & Angold, 2006; Wichstrøm et al., 2012). Childhood mental illness is still often misdiagnosed and/or undertreated and treatments often fail to conform to treatment guidelines (Mash & Dozois, 2003, pp. 10–11). This background makes it understandable why *unpacking control* should engage professionals working with ND in children.

### Contributions of the unpacking control theory

The theory of *unpacking control* contributes to professions theory by fleshing out how efforts to control professional tasks and to define complex human problems plays out in the context of modern ND care pathways. Along with the second core resolution pattern, trust testing, findings suggest that the professions literature and social dilemma research are both necessary to understand what is going on in ND care pathways.

The unpacking theory further points to a specific set of professional concerns, which will be hard to ignore when restructuring ND care. The theory strongly discourages naïve attempts to remodel ND care structures based on single parameters such as available care or any one specific ideological framework. Rather, a complex set of issues should be taken into account and the inter-dependence of different care structures and professional concerns should be openly acknowledged, if success in ND care development is hoped for.

### Limitations and strengths

Limitations of this study are that most of the empirical data were collected from one region in the south of Sweden, and most of the remaining data come from two other Swedish regions. This challenges the generalizability to other contexts with different organizational and professional structures of ND care. Our theory should be regarded as a preliminary attempt to understand the complex considerations that has to be made by professionals when developing ND care pathways in a modern healthcare system. Yet, we hope that the analysis has produced a hypothesis about the status of the present ND landscape that can be useful enough. The theory has been well received and recognized when being presented to various Swedish healthcare professionals.

## Conclusions

The major problems for ND care professionals are resolved by controlling *unpacking*—defining the needs of ND patients through control over strategies, structures, and methods used to render ND comprehensible and treatable in the care pathway.

*Unpacking control's* main function is to regulate ND care complexity. Purposes of *unpacking control are to ECS* of ND patients, to promote *squeezing* of ND care resources to speed up the ND pathway, to *ideologize* ND unpacking to protect professional particularities, and eventually to *isolate* workplace unpacking to preserve accustomed structures.

This theory of *unpacking control* may prove useful to explain the action in other fields, which are also characterized by professionals trying to alleviate pressing, complex human needs while under stress.
